# Anti-Mi2 Antibody Positive Dermatomyositis With Hyper-Elevated Creatine Kinase: A Case Report

**DOI:** 10.7759/cureus.28899

**Published:** 2022-09-07

**Authors:** Ryuichi Ohta, Naho Yoshioka, Fumiko Yamane, Maika Hayashi, Chiaki Sano

**Affiliations:** 1 Community Care, Unnan City Hospital, Unnan, JPN; 2 College of Medicine, Shimane University Faculty of Medicine, Izumo, JPN; 3 Family Medicine, Shimane University Faculty of Medicine, Izumo, JPN; 4 Community Medicine Management, Shimane University Faculty of Medicine, Izumo, JPN

**Keywords:** azathioprine, immunoglobulin, anti-mi2 antibody, family medicine, rural hospital, creatine kinase, dermatomyositis

## Abstract

Dermatomyositis (DM) is a critical disease that affects the quality of life of middle-aged and older patients. The clinical findings of DM can be determined by serological profiles of autoantibodies specific to DM. We report the case of a 65-year-old female patient with anti-Mi-2 antibody-positive DM and severe muscular findings. She endured muscular symptoms for three months without appropriate treatment. The patient was successfully treated with prednisolone, azathioprine, and intravenous immunoglobulins. This case highlights the importance of intensive treatment of DM with extremely high creatine kinase levels with steroids, immunosuppressants, and immunoglobulin treatments and the necessity of education on help-seeking behaviors in dealing with symptoms among rural older people to prevent the progression of autoimmune diseases and treat them at an early stage.

## Introduction

Dermatomyositis (DM) is a critical disease affecting the quality of life of middle-aged and older patients. The pathophysiology of this disease is explained by autoimmunity mainly against the muscles and skin. In critical cases, inflammation spreads to the lungs and kidneys, which may cause interstitial pneumonia and acute nephritis with high mortality [1,2]. Advancements in treatment with steroids, immunosuppressants, and intravenous immunoglobulin therapy can mitigate the severity of DM symptoms [3]. For an effective diagnosis, a precise clinical history and physical examination are essential to detect changes in the skin and nails, such as erythema on the hands and vascular dilatation in the nail folds [4]. Prompt diagnosis and treatment of DM are critical, and delaying treatment can lead to a poor prognosis.

The clinical presentations of DM can be determined by serological profiles of autoantibodies specific to DM. Autoantibodies specific to DM include anti-aminoacyl tRNA synthetase (ARS), anti-melanoma differentiation-associated gene 5 (MDA5), anti-transcriptional intermediary factor 1γ (TIF1γ), and anti-Mi-2 antibodies [5]. Patients with DM could show specific symptoms with each antibody, such as anti-ARS antibody showing interstitial pneumonia, anti-MDA5 antibody showing diffuse alveolar damage, anti-TIF1γ antibody showing coexistence of malignancy, and anti-Mi-2 antibody showing muscular damage [5]. However, since DM with anti-Mi-2 antibody may have clinical findings only in muscles and rarely involves the lungs, the quality of life among patients with DM may not be related to the type of autoantibodies present [6,7]. We encountered a female patient with DM specific for the anti-Mi-2 antibody and severe muscular findings. She endured muscular symptoms for three months without appropriate treatment. This case demonstrates the importance of intensive treatment of DM with extremely high creatine kinase (CK) levels and education about help-seeking behavior to manage symptoms in the rural population.

## Case presentation

A 65-year-old woman was admitted to our community hospital with the chief complaints of generalized myalgia and systemic rash for two months. Two months before admission, she noticed generalized muscle pain and rash on the extensor parts of her hands after picking up the leaves of a Japanese pepper on a nearby mountain. She visited a dermatologist and was treated with steroid ointment. One month before admission, she experienced pain in several joints of her hands and nails. Twenty days before admission, the patient experienced worsening generalized muscle pain. She visited a rural orthopedic clinic and was prescribed acetaminophen of 500mg. Her symptoms had not been alleviated with medication; therefore, she visited a primary care doctor four days before admission and was observed without medication. The rash had spread to her thighs, neck, and eyebrows. On the day of admission, the patient was referred to our hospital for further evaluation by the primary care doctor. Her medical history included hypertension, dyslipidemia, brain infarction, and reflux esophagitis. Her medication regimen included benidipine (4 mg/day).

**Figure 1:**
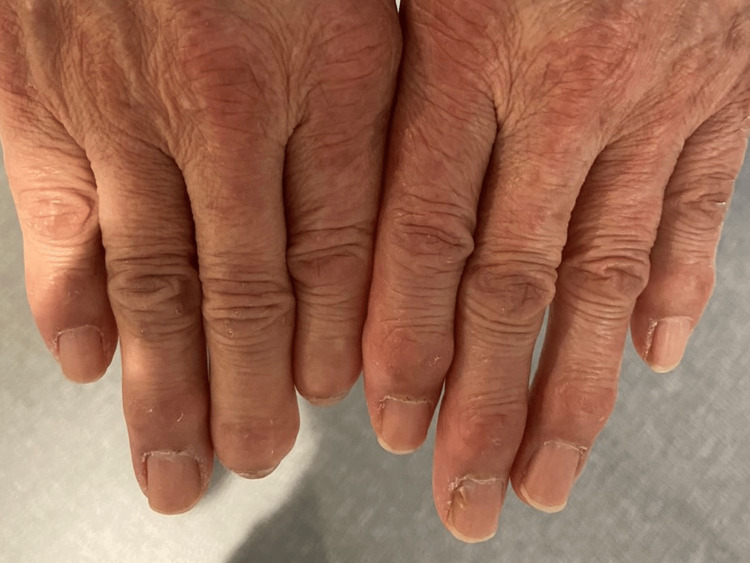
Gottron sign on both hands

On admission, her vital signs were as follows: blood pressure, 133/82 mmHg; pulse rate, 102 beats/min; respiratory rate, 18 breaths/min; body temperature, 36.7°C; and SpO2, 97% on room air. On the physical examination, Gottron signs were observed on the extensor side of the hands (Figure 1).

**Figure 2:**
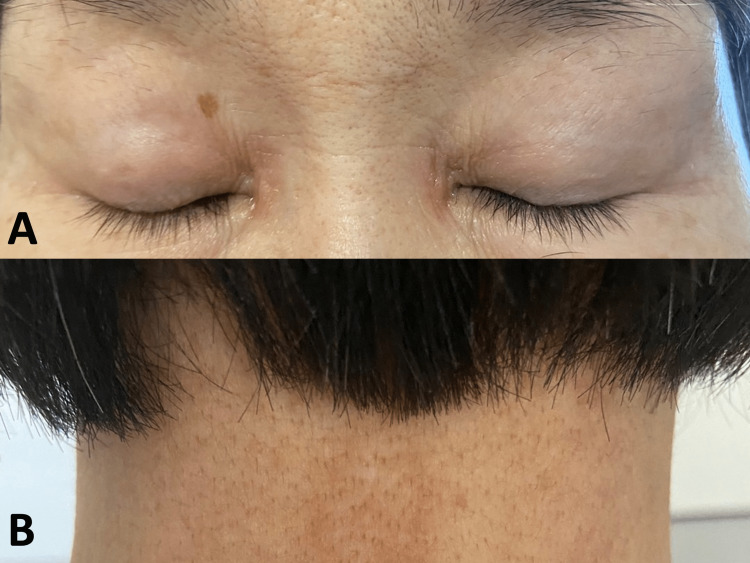
Heliotrope rash on her eyelids (A) and crusts on the posterior neck (B)

She had erythema, crusts on the precordium and posterior neck, and a heliotrope rash on her eyelids (Figure 2).

She had tenderness in the bilateral detroid, biceps brachii, and quadriceps. Regarding the manual muscle test, all of the muscles were normal. A physical examination of the chest, heart, and abdomen revealed no abnormalities. Laboratory tests showed abnormal elevations in liver enzymes and CK levels (Table 1).

**Table 1:** Initial laboratory data of the patient

Marker	Level	Reference
White blood cells	4.4	3.5–9.1 × 10^3^/μL
Neutrophils	70.9	44.0–72.0%
Lymphocytes	18.8	18.0–59.0%
Monocytes	5.9	0.0–12.0%
Eosinophils	3.4	0.0–10.0%
Basophils	1.0	0.0–3.0%
Red blood cells	4.15	3.76–5.50 × 10^6^/μL
Reticulocytes (%)		/μL (%)
Hemoglobin	13.1	11.3–15.2 g/dL
Hematocrit	40.1	33.4–44.9%
Mean corpuscular volume	96.8	79.0–100.0 fl
Platelets	29.9	13.0–36.9 × 10^4^/μL
Erythrocyte sedimentation rate	58	2–10 mm/hour
Total protein	6.2	6.5–8.3 g/dL
Albumin	3.2	3.8–5.3 g/dL
Total bilirubin	0.3	0.2–1.2 mg/dL
Aspartate aminotransferase	406	8–38 IU/L
Alanine aminotransferase	222	4–43 IU/L
Alkaline phosphatase	71	106–322 U/L
γ-Glutamyl transpeptidase	14	<48 IU/L
Lactate dehydrogenase	965	121–245 U/L
Blood urea nitrogen	17.1	8–20 mg/dL
Creatinine	0.49	0.40–1.10 mg/dL
eGFR	90≦	>60.0 mL/min/L
Serum Na	143	135–150 mEq/L
Serum K	3.8	3.5–5.3 mEq/L
Serum Cl		98–110 mEq/L
Serum Ca	108	3.5–5.3 mg/dL
Serum P	2.8	0.2–1.2 mg/dL
Serum Mg	2.2	1.8–2.3 mg/dL
Ferritin	471.1	14.4–303.7 ng/mL
CK	17495	56–244 U/L
CRP	0.46	<0.30 mg/dL
TSH	2.17	0.35–4.94 μIU/mL
Free T4	0.8	0.70–1.48 ng/dL
IgG	1464	870–1700 mg/dL
IgM	95	35–220 mg/dL
IgA	170	110–410 mg/dL
IgE	222	<173 mg/dL
HBs antigen	0.00	IU/mL
HBs antibody	0.28	mIU/mL
HBc antibody	0.12	S/CO
HCV antibody	0.00	S/CO
Syphilis treponema antibody	0.0	S/CO
SARS-CoV-2 antigen	-	
Urine test		
Leukocyte	(-)	
Nitrite	(-)	
Protein	(+-)	
Glucose	(-)	
Urobilinogen	NORMAL	
Bilirubin	(-)	
Ketone	(-)	
Blood	3+	
Rheumatoid factor	18	
Antinuclear antibody	1280≦	
SPECKLED	1280≦	
C3	133	
C4	32	
KL-6	255	
Anti SS-A antibody	<1.0	
Anti SS-B antibody	<1.0	
Anti Jo-1 antibody	(-)	
Anti-Cyclic citrullinated peptide antibody	<0.6	
Anticardiolipin antibody	<4.0	
Anti-melanoma differentiation-associated gene5 antibody	(-)	
Anti-Mi-2 antibody	≧150	
Anti-Transcriptional intermediary factor 1γ antibody	(-)	

Chest radiography revealed no interstitial opacity. Chest and abdominal computed tomography revealed no obvious lymphadenopathy or mass lesion. The patient was diagnosed with DM with elevated myogenic enzyme levels. 

**Figure 3:**
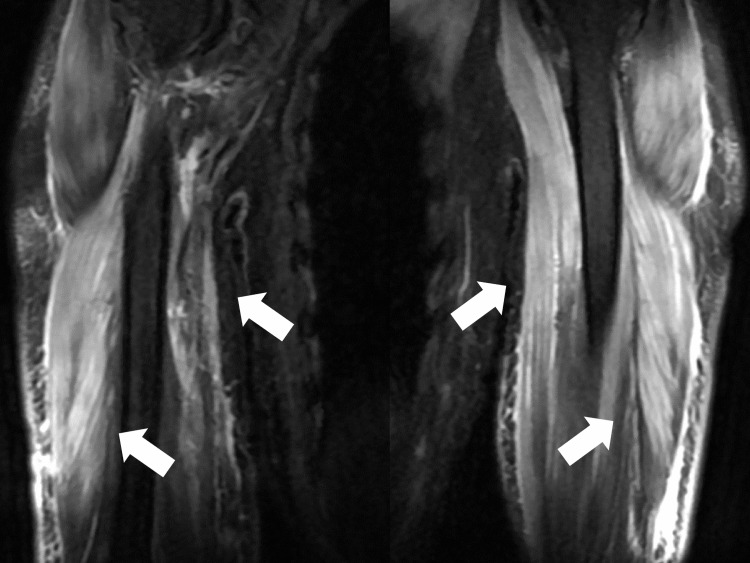
Magnetic resonance imaging (fat suppression images) of both arms shows high signal in the entire muscles of both arms (white arrows)

A three-day course of methylprednisolone (1000 mg) was administered intravenously on the first day of admission for acute exacerbation of DM. On the fourth day of admission, prednisolone 50 mg was started with sulfamethoxazole and trimethoprim. On the same day, the antinuclear antibody blood test results showed the presence of an anti-Mi-2 antibody. On the sixth day of admission, the CK levels decreased to 6000 U/L. Fluid replacement therapy was initiated to consider the possibility of renal damage due to rhabdomyolysis. Magnetic resonance imaging (fat-suppression images) of both arms showed a high signal intensity in all muscles of both arms (Figure 3).

Azathioprine (25 mg) was initiated to enhance immunosuppression and increased to 50 mg one week later. CK did not decrease as observed by blood sampling, and the second cycle of steroid pulse therapy was started on day eight of enrolment.

On the 14^th^ day of admission, the laboratory test results showed sustained CK levels of 5000 U/L. Intravenous immunoglobulin (0.4 mg/kg/day) was started for five days, and a decrease in CK to 4000 was confirmed by blood sampling in the following days. Her muscle pain and general fatigue were alleviated, and she was discharged on the 21^st^ day of admission. At discharge, prednisolone was tapered to 30 mg per oral, and azathioprine was increased to 75 mg. Her symptoms improved during her outpatient follow-up in the outpatient department, and she was able to resume normal activities.

## Discussion

This case report describes an elderly female patient with DM who tested positive for Mi2 antibody and had extremely high CK level. This case highlights the importance of intensive treatments for DM with extremely high CK with steroids, immunosuppressants, and immunoglobulins, as well as the need to educate about help-seeking behaviors in managing symptoms in rural elderly to prevent the progression of autoimmune diseases and to treat them early.

Severe cases of DM may have sustained clinical courses with high CK levels, requiring intensive treatment with steroids, immunosuppressants, and immunoglobulins to reduce the risk of various complications. In this case, an initial CK level of > 10000 U/L can be a risk factor for rhabdomyolysis, leading to acute renal injury [8,9]. The treatment of rhabdomyolysis is usually intensive hydration with extracellular fluid [8,9]. As one of the etiologies of rhabdomyolysis is DM, intensive treatment with steroid pulse therapy, immunosuppressants, and immunoglobulin infusion is needed to suppress the immunological reactions that destroy muscles. Previous studies have reported critical cases of high creatine and interstitial pneumonia in DM with intensive treatment for interstitial pneumonia [10,11]. In contrast, anti-Mi2 antibody-positive DM mainly causes an extreme increase in CK levels without malignancy or interstitial pneumonia [12,13]. Therefore, to effectively treat DM with high CK levels, prompt suppression of immunological reactions by multiple treatments is essential to prevent rhabdomyolysis complications.

Delays in treating anti-Mi2 antibody-positive DM can cause poor prognosis; therefore, effective collaboration among medical professionals and appropriate help-seeking behaviors of patients is crucial [14]. In this case, the patient visited our hospital two months after the appearance of symptoms. DM progression over two months could make treatment difficult due to muscle inflammation. Early treatment of DM may provide a better clinical course for patients [12]. Ant-Mi2 antibody-positive DM induces strong muscle inflammation compared with other autoantibodies related to DM [12]. In a few months without treatment, patients with ant-Mi2 antibody positive DM may experience deterioration of their daily living activities. Detecting DM in primary care and consulting with hospitals is essential for effective treatment.

Collaboration and education among patients and healthcare professionals are essential for effective treatment of autoimmune diseases in rural contexts. Considering this case and rural medicine, rural healthcare resources should be effectively used in treating autoimmune diseases [15]. In this case, a smooth collaboration among primary care clinics could have detected DM, leading to prompt general hospital consultation and effective treatment. In addition, older patients’ help-seeking behaviors should be enhanced to ensure smooth collaboration between rural clinics and hospitals [16]. The patient, in this case, visited several clinics without information continuity, which could have been the reason for the delayed diagnosis. Self-management and self-medication can be related to the quality of life among older rural people [17,18]. Rural older people should be educated on effectively using their resources, such as their knowledge, over-the-counter drugs, and rural healthcare resources [19].

## Conclusions

This case highlights the clinical importance of intensive treatments for DM with extremely high CK levels with steroids, immunosuppressants, and immunoglobulins at early stages. Rural older adults should be educated on the importance of seeking help when they experience symptoms to prevent the progression of autoimmune diseases and treat them in the initial stage, in addition to self-management and self-medication.
